# Longitudinal Trend of Health-Related Quality of Life During Concurrent Chemoradiotherapy and Survival in Patients With Stage II–IVb Nasopharyngeal Carcinoma

**DOI:** 10.3389/fonc.2020.579292

**Published:** 2020-10-08

**Authors:** Ji-Bin Li, Shan-Shan Guo, Lin-Quan Tang, Ling Guo, Hao-Yuan Mo, Qiu-Yan Chen, Hai-Qiang Mai

**Affiliations:** ^1^Department of Clinical Research, Sun Yat-sen University Cancer Center, Guangzhou, China; ^2^State Key Laboratory of Oncology in South China, Guangdong Key Laboratory of Nasopharyngeal Carcinoma Diagnosis and Therapy, Collaborative Innovation Center for Cancer Medicine, Sun Yat-sen University Cancer Center, Guangzhou, China; ^3^Department of Nasopharyngeal Carcinoma, Sun Yat-sen University Cancer Center, Guangzhou, China

**Keywords:** longitudinal trend, health-related quality of life, cheomotherapy, nasopharyngeal carcinoma, radiotherapy

## Abstract

**Background and Aims:** To investigate the longitudinal trend of health-related quality of life (HRQOL) from the start to the end of concurrent chemoradiotherapy and survival in patients with advanced nasopharyngeal carcinoma (NPC).

**Methods:** A total of 145 patients with stage II–IVb NPC, who were a subsample of a randomized phase III clinical trial, were recruited in this study. HRQOL was measured weekly for a total of 6 weeks during concurrent chemoradiotherapy by the Chinese version of the European Organization for Research and Treatment of Cancer Quality of Life Questionnaire core 30. Longitudinal trends of HRQOL domains over time were analyzed using mixed models. Survival rates were estimated using Kaplan-Meier method.

**Results:** During a median follow-up of 45 months, the 3-year progression-free survival rate, overall survival rate, and distant metastasis-free survival rate were highly at 86.8% (95% CI: 80.1%, 91.4%), 95.1% (95% CI: 90.1%, 97.6%), and 91.0% (95% CI: 84.9%, 94.6%), respectively. The average weekly declines of five functioning domains were 1.83–3.52 points during the treatment period, with role functioning having the largest decline rate (−2.52 points per week, 95% CI: −4.50, −2.55; *p* < 0.001). Loss of appetite is the most affected symptom, with severe appetite loss ranging from 35.9 to 61.1%. The average increases of symptoms were 0.63–5.16 points per week during treatment period (all *p*-values for time <0.001, except for financial difficulties), with pain symptoms having the largest increase (5.16 points, 95%CI: 4.25, 6.08; *p* < 0.001), followed by fatigue (3.62 points, 95%CI: 2.90, 4.35; *p* < 0.001).

**Conclusion:** The HRQOL of patients with advanced NPC is poor and substantially deteriorated during the concurrent chemoradiotherapy (CCRT) period. Psychological care and support is necessary for patients with advanced NPC during the treatment period.

## Introduction

Nasopharyngeal carcinoma (NPC) is a malignant tumor arising from nasopharynx epithelium with an extremely unbalanced geographical global distribution. There were about 129,000 new cases worldwide in 2018, with more than 70% of new cases in East and Southeast Asia (1, 2). In China, the world age-standardized incidence rate of NPC was 2.17/100,000, and the highest rate was observed in Southern China from Guangdong and Guangxi province (3, 4). Most patients with NPC were in stage II–IVb at initial diagnosis. Concurrent chemoradiotherapy (CCRT), recommended by the National Comprehensive Cancer Network, is the standard treatment for patients with stage II–IVb NPC (5). Some studies have indicated that the local control rate and 5-year overall survival rate of patients with NPC are up to 90 and 80%, respectively (6, 7). However, the complications and treatment-related adverse effects are still non-ignorable. Patients with NPC are significantly affected by difficulties in swallowing, hearing loss, xerostomia, speech impediments, and psychological issues (e.g., depression, anxiety), which further aggravate their health-related quality of life (HRQOL) (8, 9).

The majority of previous studies mainly focused on the endpoints of overall survival, progression-free survival, or local control rate from the physician's point of view. In recent years, HRQOL has been recognized as an important treatment endpoint from patients' experience to perform a comprehensive evaluation and has been increasingly used in oncology trials for clinical decision making (10). HRQOL is an important outcome for patients with head and neck cancers, especially for NPC. The diagnosis and treatment of NPC is a life-threatening event, and patients with locally advanced NPC experience distressing issues such as pain, loss of appetite, and impairment in social and role functioning in terms of HRQOL at diagnosis (11). These problems are common not only at diagnosis and during treatment, but also for several years after (12). Pretreatment HRQOL has been reported as a predictor of survival for patients with NPC (13, 14) and other advanced cancers [e.g., lung cancer (15), breast cancer (16), colorectal cancer (17), and hepatocellular carcinoma (18)]. A change in scores of many HRQOL domains from initial to 6 months after radiation therapy has been significantly associated with overall survival in patients with head and neck cancers (19). Results has also shown that HRQOL (i.e., physical functioning, fatigue, appetite loss) after treatment significantly predicted disease-free survival and overall survival in patients with NPC (20). In addition, it was found that global quality of life, insomnia, and fatigue were significant predictors of weight loss (21), which has been associated with poor survival of patients with NPC (22, 23). Therefore, maintaining a high level of HRQOL during the treatment period is important for patient prognosis and psychological well-being. It has been reported that advanced NPC patients who received CCRT reported worse HRQOL compared to those who received radiotherapy (24). HRQOL in head and neck cancer patients deteriorates immediately after treatment and then gradually recovers to pretreatment levels at around 12 months after treatment (25). To the best of our knowledge, the variation trend of HRQOL during the CCRT period has not yet been established among patients with NPC. Such evidence may be helpful for physicians to act preventively or come up with recommendations for improving HRQOL in patients with NPC during and after the treatment period.

In this study, we explored the longitudinal trend of HRQOL during the CCRT period and survival among patients with II–IVb NPC, using the longitudinal data from a randomized phase III clinical trial (26). It is hypothesized that HRQOL would gradually deteriorate during the CCRT period.

## Methods

### Study Design and Participants

Participants in this study were a subsample from a multicenter, open label, non-inferiority, randomized phase III trial (26). In the trial, between 16 January 2012 and 16 July 2014, a total of 402 patients aged 18–65 years with stage II–IVb NPC, a Karnofsky score of ≥70, and adequate hematological, renal, and hepatic function were randomly assigned (1:1) to intravenously receive either nedaplatin (100 mg/m^2^) or cisplatin (100 mg/m^2^) on days 1, 22, and 43 for three cycles concurrently with intensity-modulated radiotherapy. The exclusion criteria included previous radiotherapy or chemotherapy for NPC; the presence of relapse or distant metastasis; a previous malignancy (apart from carcinoma *in situ* of the cervix, or basal or squamous cell carcinoma of the skin); the presence of uncontrolled life-threatening illness; pregnancy or lactation; and any mental disorder or somatic comorbidities of clinical concern.

Among the 402 randomized patients, 145 (36%) returned completed the European Organization for Research and Treatment of Cancer Quality of Life Questionnaire core 30 (EORCT QLQ-C30) surveys at baseline (before radiotherapy), making them suitable for the current study. The study was approved by the ethics committee or institutional review board at each participating center, and all patients provided written consent.

### Data Collection Procedures

All patients were randomly assigned to receive intravenous nedaplatin or nedaplatin (100 mg/m^2^) on days 1, 22, and 43 for three cycles concurrently with intensity-modulated radiotherapy (2·00–2 33 Gy per fraction with five daily fractions per week for 6–7 weeks) (26).

HRQOL was assessed using the EORTC QLQ-C30, version 3.0 (27). Its Chinese version has been validated in a previous study (28). The EORTC QLQ-C30 is a 30-item generic cancer instrument which evaluates a global quality of life (QoL), five multi-item functioning scales (i.e., physical, role, emotional, cognitive, and social functioning), three multi-item symptom scales (fatigue, pain, and nausea/vomiting), and six single symptom items (dyspnea, insomnia, appetite loss, constipation, diarrhea, and financial difficulties). HRQOL scales were summarized as standard scores ranging from 0 to 100 according to the scoring manual (29). A higher score for global QoL and functioning scales represents a better level of global QoL or functioning, whereas a higher score for symptom scales/items indicates a higher level of symptomatology/problems.

EORTC QLQ-C30 was self-administered weekly for a total of 6 weeks during the CCRT period. All assessments were carried out by a well-trained clinical research coordinator at the clinics. Sociodemographic characteristics were collected at the recruitment interview. After completion of treatment, participants were followed up at least every 3 months during the first 3 years and every 6 months thereafter until death. Progression-free survival was assessed by the investigator and defined as the time from the date of randomization to documented local or regional relapse, distant metastasis, or death from any cause, whichever occurred first. Overall survival was defined as the time from the date of randomization to death from any cause or censored at the date of last follow-up. Distant metastasis-free survival was defined as the time from the date of randomization to distant metastasis, or death from any cause. The censored date of the study was 31 June 2017.

### Statistical Analysis

Descriptive analysis was presented as mean with standard deviation or frequency with percentage when appropriate. The longitudinal trend of the HRQOL scale scores from the beginning to the end of treatment was analyzed with mixed models using restricted maximum likelihood estimation and an unstructured covariance structure. Each mixed model included one of the HRQOL domains as a dependent variable, an intercept, and an independent variable representing time points during CCRT period, by univariable and adjustment of sociodemographic and clinical covariates separately. Two random effects were included in the mixed models: a random patient effect representing an individual baseline HRQOL (intercept) and a random subject by time effect respecting an individual linear change per week during the treatment period (slope of time variable). Regression coefficients along with 95% confidence interval (95% CI) of time were reported.

Given that scores of ≤50 for global QoL and functioning scales or scores of >50 for symptom scales/items indicate a need for intervention (30), we applied an absolute threshold value of 50 points for describing very low global QoL and functioning scores as worse global QoL / functioning or very high symptom scores as severe symptoms. The distributions of worse HRQOL were presented stratified by measurement time points.

All statistical analyses were performed using SAS for windows version 9.4 (SAS Institute, Cary, NC, USA). A two-sided *p* < 0.05 was considered statistically significant. The original randomized phase III clinical trial is registered on ClinicalTrials.gov with number NCT01540136 (26). The key raw data of this study have been uploaded onto the Research Data Deposit platform (RDD), with approval number RDDA2018000932.

## Results

### Social-Demographic and Clinical Characteristics

Among 145 patients at baseline, 51.7% received cisplatin-based CCRT, and 48.3% received nedaplatin-based CCRT. The mean age of the patients was 44.3 years old (standard deviation: 9.8), 74.5% of whom were male, 36.6% had a history of smoking, and 24.8% had a history of drinking. Around 24.2% of patients were overweight or obese at baseline, and more than half of patients (57.3%) were observed to experience 5% or more of weight loss during the treatment period as compared to their baseline bodyweight (Table 1).

**Table 1:** Sample characteristics.

	***n***	**%**
Age at randomization, years, mean ± SD	44.3 ± 9.8	
≤45	80	55.2
>45	65	44.8
Sex		
Male	108	74.5
Female	37	25.5
Smoking habit		
No	92	63.4
Yes	53	36.6
Drinking habit		
No	109	75.2
Yes	36	24.8
BMI, kg/m^2^, mean ± SD	23.0 ± 2.9	
<18.5	6	4.1
18.5–24.9	104	71.7
25.0–29.9	34	23.5
≥30	1	0.7
Percentage of weight loss during CCRT period, mean ± SD	−6.7% ± 6.6%	
No change or increase	25	17.2
<5%	37	25.5
5–10%	41	28.3
>10%	42	29.0
T stage		
T1	1	0.7
T2	27	18.6
T3	90	62.1
T4	27	18.6
N stage		
N0	14	9.7
N1	56	38.6
N2	66	45.5
N3	9	6.2
AJCC stage		
II	15	10.5
III	95	65.5
IV	35	24.0
Epstein-Barr virus DNA test		
DNA <1,500 copies per mL	79	54.5
DNA ≥1,500 copies per mL	66	45.5
Intervention		
Cisplatin	75	51.7
Nedaplatin	70	48.3
Chemotherapy cycles		
Two or less	39	26.9
Three	106	73.1
Duration of radiotherapy		
≤42 days	55	37.9
>42 days	90	62.1

*CCRT, concurrent chemoradiotherapy; AJCC, American joint committee on cancer; SD, standard deviation*.

### Treatment Completion

All of 145 patients completed the recommended radiotherapy. The median dose of RT was 70 Gy (Range: 70–70 Gy), and the median dose per fraction was 2.33 Gy (Range: 2.12–2.33 Gy). Almost all patients (99.3%, 144/145) received at least two cycles of chemotherapy, with 73.1% (106/145) of patients completed the three cycles. Besides, 38.6% (56/145) received chemotherapy with dosage 300 mg/m^2^, and 93.8% (136/145) patients received chemotherapy with dosage ≥200 mg/m^2^.

### Survival Rate

**Figure 1:**
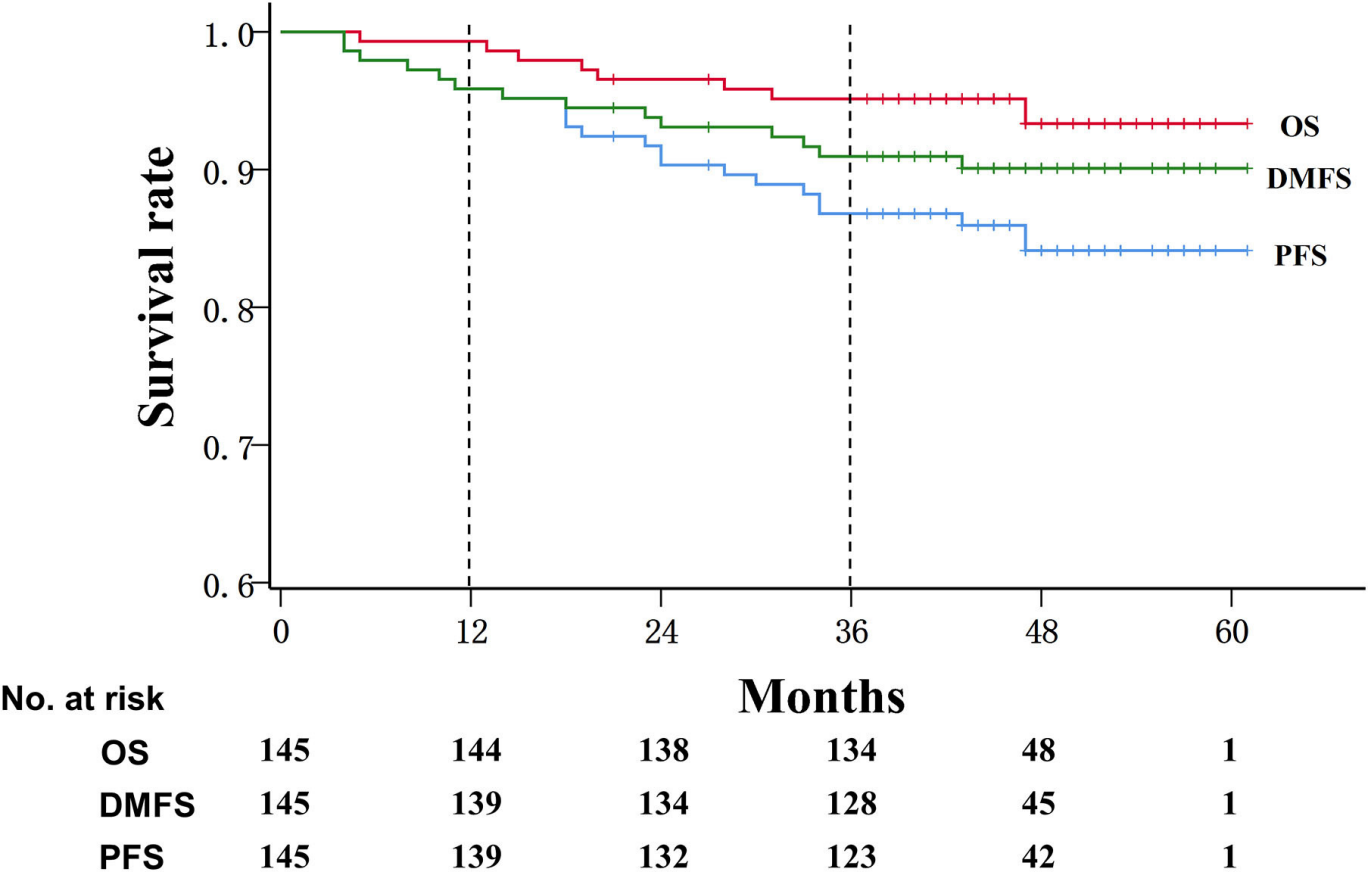
Kaplan–Meier survival curve in patients with advanced nasopharyngeal carcinoma. OS, overall survival; DMFS, Distant metastasis-free survival; PFS, progression-free survival.

During a median follow-up of 45 months, the progression-free survival rate was 95.9% (95% CI: 91.0, 97.7%) at 1 year and 86.8% (95% CI: 80.1, 91.4%) at 3 years, and the overall survival rate was 99.3% (95% CI: 95.2, 99.9%) at 1 year and 95.1% (95% CI: 90.1, 97.6%) at 3 years, whereas the distant metastasis-free survival rate was 95.9% (95% CI: 91.0, 98.1%) at 1 year and 91.0% (95% CI: 84.9, 94.6%) at 3 years (Figure 1).

### HRQOL Completion

Of 145 patients who completed baseline surveys, 96 (66.2%), 139 (95.9%), 106 (73.1%), 128 (88.2%), and 144 (99.3%) provided valid data at week 2 to week 6 during the CCRT period, respectively. The attrition rate was the greatest at week 2 of the CCRT period. Participants were more likely to miss EORTC QLQ-C30 questionnaires at week 2 if they received two or less cycles of chemotherapy, were progression-free, were alive or were distant metastasis-free during the study period, while there were no significant differences of other sample characteristics between participants with and without EORTC QLQ-C30 questionnaires at week 2 (see online Supplementary Table 1). A total of 62.8% of patients completed all six-point assessments, 89.7% patients completed more than three assessments, and only 2.1% missed four of six assessments during the treatment period.

### Trend of Global QoL and Functioning Domains

**Figure 2:**
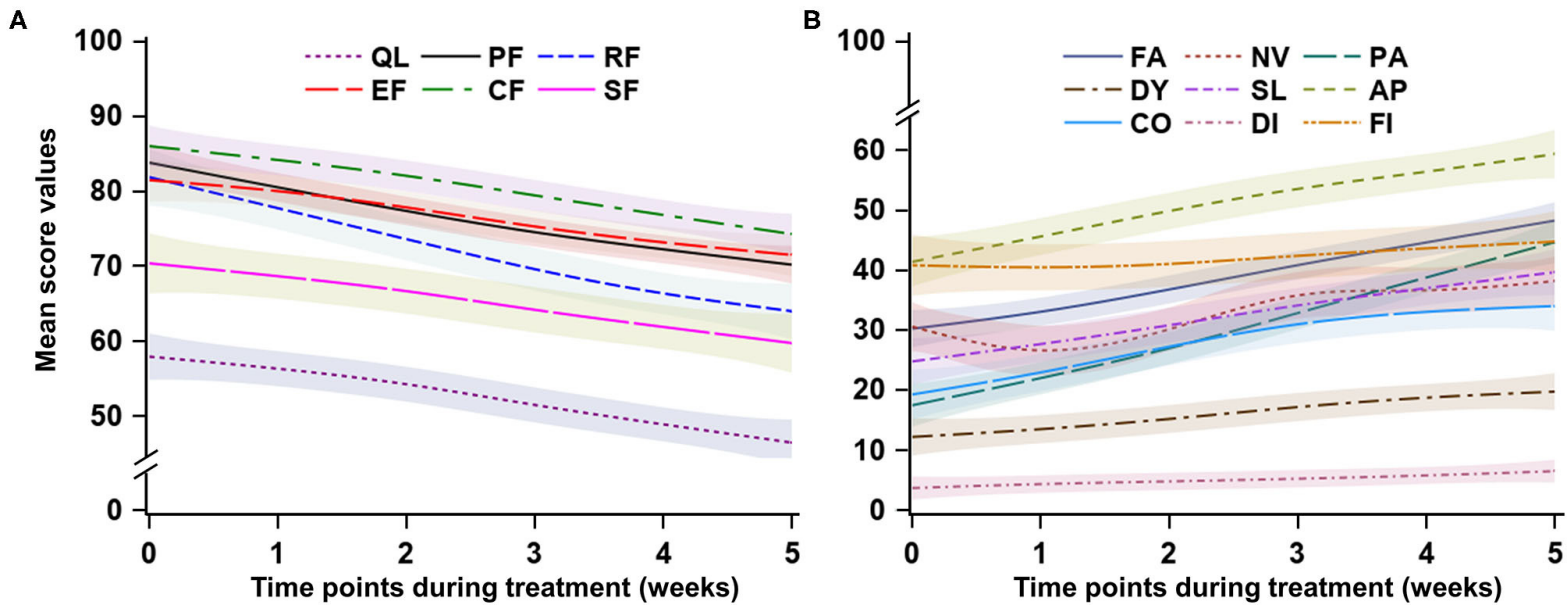
Trend of health-related quality of life during the concurrent chemoradiotherapy period. QL, global quality of life; PF, physical functioning; RF, role functioning; EF, emotional functioning; CF, cognitive functioning; SF, social functioning; FA, fatigue; NV, nausea and vomiting; PA, pain; DY, dyspnea; SL, sleep disturbance; AP, appetite loss; CO, constipation; DI, Diarrhea; FI, financial difficulties. For **(A)**, a higher score represents better quality of life or functioning; for **(B)**, a higher score represents more severe symptoms.

After adjusting the social-demographic and clinical characteristics listed in Table 1, the mixed models indicated a substantial deterioration across the 6-week treatment period in global QoL and five functioning domains. The global QoL had the lowest values compared to the five functioning domains in all six time points from 57.6 to 46.6, with an average decline of 2.18 points per week (95% CI: −3.07, −1.30). (Table 2 and Figure 2A).

**Table 2:** Temporal trend of health-related quality of life (HRQOL) during the concurrent chemoradiotherapy period using the mixed model.

	**Measurement time points**						**Regression coefficient (95% CI)**	
	**Week 1**	**Week 2**	**week 3**	**Week 4**	**Week 5**	**Week 6**	**Time^***a***^**	**Time^***b***^**
Global quality of life^†^	57.6 ± 21.5	56.2 ± 21.5	56.1 ± 21.0	49.4 ± 19.4	49.2 ± 21.4	46.6 ± 22.1	−2.19 (−3.07, −1.30)^***^	−2.18 (−3.07, −1.30)^***^
Psychical function^†^	84.0 ± 13.8	80.6 ± 15.4	77.7 ± 17.8	73.4 ± 17.4	72.7 ± 19.5	70.4 ± 19.4	−2.73 (−3.36, −2.09)^***^	−2.72 (−3.36, −2.08)^***^
Role function^†^	81.9 ± 22.3	77.6 ± 24.4	74.6 ± 25.2	68.7 ± 26.5	65.2 ± 27.6	64.9 ± 28.3	−3.52 (−4.49, −2.55)^***^	−3.52 (−4.50, −2.55)^***^
Emotional function^†^	80.7 ± 17.2	81.8 ± 18.5	78.5 ± 18.6	73.9 ± 20.7	72.9 ± 21.5	72.0 ± 20.4	−1.83 (−2.41, −1.25)^***^	−1.83 (−2.41, −1.26)^***^
Cognitive function^†^	85.9 ± 15.9	83.9 ± 15.4	83.3 ± 16.6	78.3 ± 19.4	77.0 ± 21.5	74.4 ± 21.6	−2.21 (−2.85, −1.57)^***^	−2.21 (−2.85, −1.57)^***^
Social function^†^	70.2 ± 26.9	68.2 ± 26.7	68.3 ± 26.4	62.3 ± 28.6	62.2 ± 28.1	59.8 ± 27.9	−2.05 (−2.86, −1.23)^***^	−2.06 (−2.88, −1.25)^***^
Fatigue ^‡^	31.3 ± 20.6	30.3 ± 19.2	37.0 ± 21.2	42.1 ± 22.4	44.4 ± 22.4	48.2 ± 21.8	3.64 (2.92, 4.36)^***^	3.62 (2.90, 4.35)^***^
Nausea and vomiting^‡^	31.3 ± 28.4	25.0 ± 21.9	30.0 ± 26.9	37.7 ± 26.3	35.7 ± 24.8	38.4 ± 25.5	2.26 (1.35, 3.18)^***^	2.25 (1.34, 3.16)^***^
Pain^‡^	17.8 ± 19.3	22.7 ± 23.1	24.9 ± 22.4	34.9 ± 26.1	38.5 ± 27.6	44.8 ± 27.4	5.17 (4.25, 6.08)^***^	5.16 (4.25, 6.08)^***^
Dyspnea^‡^	12.6 ± 20.1	12.8 ± 19.6	14.4 ± 19.7	18.2 ± 23.5	19.5 ± 23.5	19.2 ± 21.4	1.43 (0.68, 2.18)^***^	1.44 (0.69, 2.19)^***^
Sleep disturbance^‡^	25.1 ± 25.6	27.4 ± 24.7	30.0 ± 26.1	35.8 ± 27.9	36.7 ± 27.4	39.6 ± 27.6	2.94 (1.93, 3.94)^***^	2.93 (1.93, 3.94)^***^
Appetite loss^‡^	41.6 ± 30.3	44.4 ± 27.6	50.1 ± 26.7	56.0 ± 27.8	54.7 ± 26.4	59.7 ± 27.8	3.60 (2.50, 4.71)^***^	3.61 (2.50, 4.72)^***^
Constipation^‡^	20.2 ± 26.1	19.4 ± 24.0	27.6 ± 28.1	33.6 ± 27.0	33.3 ± 31.9	33.1 ± 30.9	2.71 (1.45, 3.96)^***^	2.69 (1.44, 3.94)^***^
Diarrhea^‡^	3.4 ± 10.9	5.2 ± 14.0	4.6 ± 12.2	5.3 ± 13.9	5.5 ± 14.4	6.7 ± 14.5	0.63 (0.21, 1.06)^***^	0.63 (0.21, 1.06)^***^
Financial difficulties^‡^	41.8 ± 35.1	38.9 ± 33.4	40.0 ± 33.4	44.3 ± 36.7	43.5 ± 34.6	44.8 ± 35.8	0.43 (−0.31, 1.17)	0.43 (−0.30, 1.17)

*95% CI: 95% confidence interval*.

† *Scale 0–100: higher score represents better quality of life*.

‡ *Scale 0–100: higher score represents more severe symptoms*.

a *regression coefficients obtained by univariable mixed models*.

b *regression coefficients obtained by mixed models after adjusting of age (≤45 vs. >45), sex (male vs. female), smoking habit (no vs. yes), drinking habit (no vs. yes), BMI (<25 kg/m^2^ vs. ≥25 kg/m^2^), weight loss during CCRT (no change/increase, <5%, 5–10%, >10%), T stage (T1–2, T3, T4), N stage (N0–1 vs. N2–3), AJCC stage (II, III, IV), pretreatment Epstein–Barr virus DNA copies (<1,500 vs. ≥1,500), intervention arm (cisplatin vs. nedaplatin), number of chemotherapy cycles (≤2 cycles vs. >2 cycles), and duration of radiotherapy (≤42 days vs. >42 days)*.

*** *p < 0.001*.

The average declines per week of the five functioning domains were 1.83–3.52 points across the CCRT period. Role functioning had the largest decline rate (−2.52 points per week, 95% CI: −4.50, −2.55; *p* < 0.001), followed by physical functioning (−2.72 points per week, 95% CI: −3.36, −2.08; *p* < 0.001). Cognitive functioning remained at a relatively higher level during the treatment period. Social functioning and role functioning had the lowest values compared to the other three functioning domains. (Table 2 and Figure 2A).

**Figure 3:**
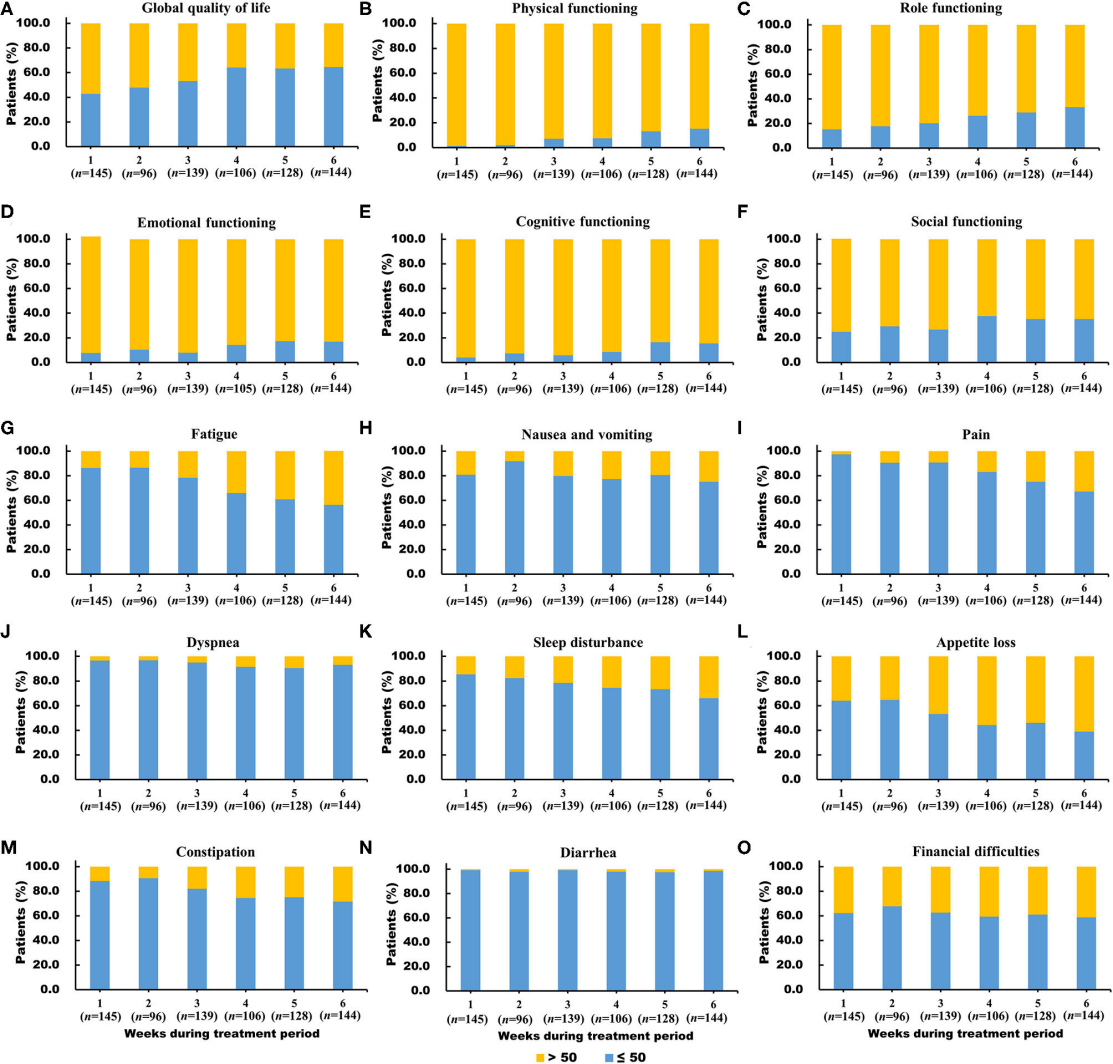
**(A-O)** Distribution of worse global quality of life (score ≤50), worse functioning (score ≤50) and severe symptoms (score >50) in the European Organization for Research and Treatment of Cancer Quality of Life Questionnaire core 30 scales during the concurrent chemoradiotherapy period.

The proportion of patients who scored ≤50 points in global QoL had a relatively high level at all six time points, increasing significantly from 42.8% at week 1 to 64.6% at week 6. The proportion of worse functioning (scores ≤50 points) increased from 1.4 to 15.3% for physical functioning, from 15.3 to 33.3% for role functioning, from 7.6 to 16.8% for emotional functioning, from 4.1 to 15.4% for cognitive functioning, and from 24.8 to 35.2% for social functioning during the treatment period (Figures 3A–F).

### Trend of Symptoms

The domain of financial difficulties remained at a stable and medium level from 41.8 points at week 1 to 44.8 points at the end of treatment, whereas the other eight symptoms significantly increased during the treatment period. The average increase of symptoms was 0.63–5.16 points per week during the treatment period (all *p*-values for time <0.001), with pain symptoms having the largest increase (5.16 points, 95% CI: 4.25, 6.08; *p* < 0.001), followed by fatigue (3.62 points, 95% CI: 2.90, 4.35; *p* < 0.001). The diarrhea symptom had the lowest values in the nine symptom domains during the treatment period, with a slight but significant increase from 3.4 to 6.7 (*p* < 0.001). The first three prominent symptoms were loss of appetite, financial difficulties, and fatigue (Table 2 and Figure 2B).

The proportion of patients with severe diarrhea symptoms (from 0.7 to 1.4%) and dyspnea (from 3.4 to 6.9%) were small during the treatment period compared to those of the other symptoms. The proportion of patients with severe loss of appetite (from 35.9 to 61.1%), fatigue (from 13.8 to 43.8%), pain (from 2.8 to 32.9%), and sleep disturbance (from 14.5 to 34.0%) largely increased during the treatment period. (Figure 3G–O).

## Discussion

In this longitudinal study, the survival rate of patients with advanced NPC is very high, which is similar to previous studies (31, 32). We observed a substantially deteriorated trend among all domains except financial difficulties in terms of HRQOL during the CCRT period. Global QoL largely declined, with more than 64% of patients scoring a severely worse level at the end of CCRT. This indicates that CCRT significantly degrades HRQOL in patients with advanced NPC. As a radiosensitive cancer, radiation-related toxicity and complications (e.g., neuropathy, hearing loss, and xerostomia) could reduce HRQOL in patients with NPC (8, 33). Previous studies have outlined a deterioration of HRQOL during the first 3 months after the initial treatment in patients with head and neck cancers (19, 34). Therefore, more psychological care and support is necessary for patients with advanced NPC during the CCRT period. Physicians should take necessary actions to improve the HRQOL of patients with NPC during the CCRT period.

We found that social functioning and role functioning are two of the most affected functioning domains by NPC and chemoradiotherapy, with role functioning having the largest decline in the five functioning domains. The findings were consolidated by the report of Hammerlid et al. reporting that patients with NPC had the worst social and role functioning compared to those with other head and neck cancers (11). Similarly, a previous study reported that role emotional and social function, which were measured by the Shot Form 36 Health Survey Questionnaire, were lower at week 3 of radiotherapy than those before therapy (35). Social functioning was also reported as the lowest of the five functioning domains among patients with recurrent NPC (36). This might be explained by the effects of symptoms and complaints (e.g., cancer-related pain, fatigue, xerostomia) caused by radiotherapy and chemotherapy. NPC patients with severe symptoms and side effects might avoid social eating and interactions with friends/relatives, and then be vulnerable to social difficulties and interpersonal and role maladjustment.

The symptom of pain is common among head and neck cancer patients. A pooled prevalence of pain is over 50% in all cancer types, with the highest prevalence of 70% in head and neck cancer patients (37). Similarly, in our study, pain is the largest worsening symptom in NPC patients during the CCRT period, with the percentage of those experiencing severe pain sharply increasing from 2.8% at the beginning to 32.9% at the end of CCRT. Oral mucositis, which was highly prevalent among NPC patients who received radiotherapy, might be the major source of pain (38). Pain could substantially and adversely affect patients' quality of life, adherence to therapy, treatment efficacy, and satisfaction with care, and might be a clinically indicator of tumor progression (39, 40). Therefore, pain management should be vital and considered in cancer care, which could substantially improve the patient-perceived value of cancer treatment (40).

We found that loss of appetite and fatigue are two of the most severe symptoms during the CCRT treatment. Mouth dryness, tasted changes, salivary gland damage, pain, and difficulties in swallowing are common adverse effects caused by radiotherapy, which undoubtedly induce patients' loss of appetite and eating difficulties (41) and then further caused malnutrition (42). Cancer-related fatigue is significantly associated with patients' psychological distress and poor quality of life, and is a risk factor for reduced survival (43). In our study, fatigue largely deteriorated during the CCRT period. It was reported that 30 to 60% of cancer patients suffer from moderate to severe fatigue during the treatment, which may reduce treatment efficacy due to non-compliance with treatment (44).

Currently, cancer-related symptoms (i.e., pain, loss of appetite, fatigue) are still undertreated and poorly controlled in clinical practices (40, 45). The findings in our study can provide useful information for physicians conducting CCRT in patients with advanced NPC. Although the benefit of better survival for advanced NPC through CCRT has been well-confirmed in our study and previous reports, it is necessary to provide appropriate support and management for such patients to improve their HRQOL and psychological well-being during the CCRT period. These findings indicate that advanced NPC patients suffered from severe symptoms (i.e., appetite loss, fatigue, and pain) and the associated functional limitations. The deteriorating trend of HRQOL during the CCRT period could serve as an alert for physicians to provide effective and supportive care or refer patients to the appropriate services when necessary.

The main strength of our study is that HRQOL was measured weekly with 6 time points from the beginning to the end of treatment, which allowed us to explore the longitudinal trend and the changes in HRQOL during the CCRT period more precisely and robustly. Another major advantage is that the design and homogeneity of patients along with data quality are robust, as our study is based on a randomized phase III clinical trial (26). However, there are some limitations when interpreting the results in the present study. First, the sample size in this study is relatively small, and the findings is warranted for further confirmation by large-sample prospective studies. Second, due to the short-term period of follow-up in this study, only a small number of events were observed during the study period (i.e., death, disease progression, and distant metastasis), which did not allow us to estimate the potential effects of impaired HRQOL during the treatment period on subsequent survival outcomes among patients with advanced NPC. Third, it is necessary to highlight that our findings could only reflect the trend of HRQOL during the treatment period. Considering that HRQOL is highly dynamic during and after treatment, the longitudinal trend after treatment is still unclear. Fourth, there may have selection bias considering the large attrition rate. The large attrition rate might be partially attributable to the better treatment effect and lower treatment-related toxicities during the CCRT period, as these patients may feel well and did not present in the clinics for follow-up as scheduled. Hence, the assumption that missing data are missing at random might not be appropriate in this study (29). HRQOL during CCRT period among patients with advanced NPC might be underestimated.

In conclusion, our study revealed that HRQOL in patients with advanced NPC is poor and largely deteriorated during the CCRT period. Social functioning and role functioning are two of the most affected functioning domains, while loss of appetite, fatigue, and pain are the three major symptoms during the CCRT period. These findings are useful for clinicians in conducting relevant clinical treatment and in designing interventions for future studies. Longitudinal studies that measure HRQOL during and after treatment over a long time frame are highly warranted to explore the long-term trends of HRQOL and their impact on survival outcomes among patients with NPC.

## Data Availability Statement

The raw data supporting the conclusions of this article will be made available by the authors, without undue reservation.

## Ethics Statement

The studies involving human participants were reviewed and approved by the ethics committee of Sun Yet-san University Cancer Center. The patients/participants provided their written informed consent to participate in this study.

## Author Contributions

J-BL, H-QM, and S-SG: study concepts. J-BL and H-QM: study design. J-BL, S-SG, and L-QT: data acquisition. S-SG, L-QT, LG, H-YM, and Q-YC: quality control of data and algorithms. J-BL: data analysis and interpretation and statistical analysis. J-BL and S-SG: manuscript preparation. J-BL, S-SG, and H-QM: manuscript editing. H-QM: manuscript review. All authors contributed to the article and approved the submitted version.

## Conflict of Interest

The authors declare that the research was conducted in the absence of any commercial or financial relationships that could be construed as a potential conflict of interest.
