# Supplementary material for: Longitudinal Trend of Health-Related Quality of Life During Concurrent Chemoradiotherapy and Survival in Patients With Stage II–IVb Nasopharyngeal Carcinoma
Source: Front Oncol. 2020 Oct 8;10:579292. doi: 10.3389/fonc.2020.579292 (PMC7578369; doi:10.3389/fonc.2020.579292)
Supplement: Supplementary file 1 [file Table_1.pdf]

Supplementary Table 1. Differences of social-demographic and clinical characteristics between patients with and without HRQOL questionnaire at week 2.

	Patients with HRQOL questionnaire at week 2				*p* values
	No		Yes		
	n	%	*n*	%	
Age at randomization, years					
≤45	53	66.3	27	33.8	0.990
>45	43	66.1	22	33.9	
Sex					
Men	75	69.4	33	30.6	0.159
Women	21	56.8	16	43.2	
Smoking habit					
No	56	60.9	36	39.1	0.073
Yes	40	75.7	13	24.5	
Drinking habit					
No	68	62.4	41	37.6	0.091
Yes	28	77.8	8	22.2	
BMI, kg/m^2					
<18.5	4	66.7	2	33.3	0.246
18.5–24.9	66	63.5	38	36.5	
25.0–29.9	26	76.5	8	23.5	
≥30	0	0	1	100.0	
Weight loss during CCRT period					
No change or increase	19	76.0	6	24	0.533
<5%	26	70.3	11	29.7	
5–10%	25	61	16	39.2	
>10%	26	61.9	16	38.1	
T category					
T1	1	100.0	0	0	0.500
T2	15	55.6	12	44.4	
T3	60	66.7	30	33.3	
T4	20	74.1	7	25.9	
N stage					
N0	12	85.7	2	14.3	0.094
N1	40	71.4	16	28.6	
N2	37	56.1	29	43.9	
N3	7	77.8	2	22.2	
AJCC stage					
II	11	73.3	4	26.7	0.354
III	59	62.1	36	37.9	
IV	26	74.3	9	25.7	
Epstein-Barr virus DNA test					
DNA <1500 copies per mL	53	67.1	26	32.9	0.806
DNA ≥1500 copies per mL	43	65.2	23	34.8	

Intervention					
Cisplatin	46	61.3	29	38.7	0.199
Nedaplatin	50	71.4	20	28.6	
Chemotherapy cycles					
Two or less cycles	14	35.9	25	64.1	<0.001
Three cycles	82	77.4	24	22.6	
Duration of radiotherapy					
≤42 days	32	58.2	23	41.8	0.110
>42 days	64	71.1	26	28.9	
Disease progression					
Yes	19	90.5	2	9.5	0.011
No	77	62.1	47	37.9	
Death					
Yes	8	100	0	0	0.033
No	88	64.2	49	35.8	
Distant metastasis					
Yes	13	92.9	1	7.1	0.035
No	83	63.4	48	36.6	

HRQOL: Health-related quality of life; BMI: Body weight index; CCRT: Concurrent chemoradiotherapy; AJCC: American joint committee on cancer.
p values were obtained by χ^2 test or Fisher exact test.
